# Cotemporaneous Rubeola and Variola

**Published:** 1826-07

**Authors:** 


24. *Contemporaneous Rubeola and Variola.* Mr. Hunter's dogma has long been proved to have exceptions. It has not often happened, however, that two eruptive diseases, so decisive in their characters as measles and small-pox, are seen progressing together. Mr. Delagarde, of Exeter, has reported an instance of this kind, in the last volume of the Medico-chirurgical Transactions. On the 29th March, Master Brookes, aged four years, fell ill of measles, then prevalent in the city. On the 1st April, the eruption appeared—interspersed on the right cheek with a few minute pimples.

2d, Measles, raspberry-tinted, distinct, and strong. The pimples were more numerous, and on some of them were seen small vesicles. The appearance of the face was inflammatory and singular. 3d, The measles turned. The pulmonary disorder was very slight. 4th, Among the brown marbled spots, frequent after measles, appeared on the breast and arms several pustules, distinct, dimpled, enlarging, having florid bases, and becoming slightly opake. Mr. D. regarded the case as a combination of small-pox and measles, and in this opinion, he was confirmed by that of an experienced medical friend. 5th, Pustules larger—full—completely opaque. 8th, Pustules turning on the face. 10th, Pustules turning on the extremities. 11th, Crusts separating. Mr. D. inoculated a child with matter taken from these pustules, and small-pox followed

We are utterly at a loss to comprehend the meaning of the passage which follows, and which seems to involve the whole in impenetrable mystification.

"On the 8th, the day on which the pustules turned, the infant, after a slight indisposition, was covered with the rubeolar eruption. On the 15th, having completely recovered from the measles, she became very feverish. Between this and the 20th, she had several convulsions. On that day small-pox appeared. The infant not having been near any other child with that disorder, it is thus proved, not only that the eruption was variolous, but that the fever was infectious." 168.

